# Measuring a family sense of coherence: a rasch-based study extending dyadic data analyses

**DOI:** 10.1186/s12904-024-01639-5

**Published:** 2025-01-09

**Authors:** Marie-Louise Möllerberg, Kristofer Årestedt, Peter Hagell, Jeanette Melin

**Affiliations:** 1 Department of Care Science, Malmö University, Faculty of Health and Society, Malmö, Sweden; 2 RISE Research Institutes of Sweden, Division Safety and Transport, Measurement Science and Technology, Göteborg, Sweden; 3 Faculty of Health and Life Sciences, Linnaeus University, Kalmar, Sweden; 4 Department of Research, Region Kalmar County, Kalmar, Sweden; 5 The PRO-CARE Group, Faculty of Health Sciences, Kristianstad University, Kristianstad, Sweden; 6 Department of Leadership, Demand and Control, Swedish Defence University, Karlstad, Sweden

**Keywords:** Cancer, Dyads, Family, Palliative care, Psychometrics, Sense of coherencess

## Abstract

**Background:**

Family sense of coherence (FSOC) seems to reduce distress in the family and promote the well-being of the family. Therefore, getting accurate measurements for families with long-term illnesses is of particular interest. This study explores dyadic data analysis from the dyadic- and single-informant perspectives, and the measurement properties of the FSOC-S12 according to the Rasch model.

**Methods:**

Racked and stacked data from 151 dyads were analyzed according to the polytomous Rasch model.

**Results:**

Notably, both the dyadic- and single-informant perspectives (i.e., racked and stacked data set-ups) showed measurement properties with minor deviations from the Rasch model according to fit statistics. However, most items had disordered thresholds and some problems with local dependency. Item hierarchies were similar in both set-ups and there was no differential item functioning (DIF) by role from the dyadic informant perspective. Four items showed DIF by informant role in the single-informant perspective.

**Conclusions:**

Our approach to handling dyadic data has shown both strengths and limitations in the evaluation of FSOC-S12, and the understanding of FSOC as a construct from the family’s view of the family’s ability as a whole (dyadic-informant perspective) and patient’s and family member’s separate views of the family’s ability as a whole (single-informant perspective).

## Background

Living with a family member with a long-term illness at the end of life is for most people, a stressful situation [1–3]. Therefore, health and well-being are important outcomes for family members caring for a person with a long-term illness [3–5]. Sense of coherence (SOC), the core concept in Antonovsky’s salutogenic model, was developed to explain why some people remain healthy in stressful life situations. SOC consists of three interrelated concepts: comprehensibility (i.e., ability to understand the situations), manageability (i.e., access to sufficient resources to manage situations), and meaningfulness (i.e., challenges are worthy of the corresponding energy investment). Antonovsky [6] argues that SOC can be described as a health-protective behaviour pattern and an effective stress buffer, thereby influencing individuals’ ability to manage to live with long-term illness meaningfully.

SOC was initially developed at the level of the individual but has since been extended to the family level as the family sense of coherence (FSOC) construct [6]. There is growing evidence that FSOC reduces psychological distress in the family [7–9] and promotes family well-being and functioning [9–11]. For instance, proper person/family-centered care for children with cancer and their families has strengthened FSOC and thereby increased the quality of family life [12]. Therefore, identifying families with weak FSOC can be a way for healthcare professionals to identify families in need of support [7].

The Sense of Coherence Scale (SOC-S) was developed to assess SOC at an individual level [13]. Later, Antonovsky and Sourani developed the Family Sense of Coherence Scale; a long 26-item version (FSOC-S26) [14] and a short 12-item version (FSOC-S12) [15]. The FSOC-S12, which is the most commonly used, has been psychometrically evaluated using classic test theory with satisfactory results [7, 10, 16, 17]. However, these studies have some significant limitations; (i) the data were treated as independent even if they were obtained from two persons within the same family [18], and (ii) the studies did not take into account that the data were ordinal, and (iii) that the responses depend on both the item and agent attributes [19–21].

As families consist of at least two parts, dyadic studies are common in family research. Nested observations within dyads (e.g., two family members) cannot be assumed to be mutually independent as they share a common context [18]. Violating the independence assumption can create a bias in the test of statistical significance and measures of associations [22]. There are several strategies to handle nonindependence: (i) collect data from one person, (ii) collect data from two persons and treat them as if they were independent (dyadic-informant perspective), or (iii) conduct separate analyses for the two dyad members (single-informant perspective) [23]. At a theoretical level, FSOC as a construct needs to be equally informed by at least two parties in the family [14, 15]. When using the FSOC-S12, this is further referred to as the dyadic-informant perspective. However, families that may need more support can be identified if they have a low level of FSOC or when patients and family members disagree when comparing their levels of FSOC [7]. This can be done by treating family members as unique individuals and then comparing the patients’ and family members’ perspectives, referred to as the single-informant perspective. For the latter one, the power in both groups is reduced and some results might be missed.

According to Kenny et al. [23, 24], analysis of dyadic data can be summarized as shown in Table 1. In line with this table, *family as a whole* corresponds to the underpinnings of FSOC [14, 15] and can be assessed using the FSOC-S12 either from a dyadic- or single-informant perspective. However, there is no strong agreement how to handle non-independence in the psychometric literature [25].

**Table 1:** Methods of data collection and analysis relating to different family composition conceptual models

	**Individual parts**	**Dyadic parts**	**Family as a whole**
Collect data from one person	E.g. patient’s view of his/her ability in the family	E.g. patient’s view of two family members’ ability in the family	E.g. patient’s view of the family’s ability as a whole
Collect data from two (or more) persons and treat them as if they were independent (dyadic-informant perspective)	NA	E.g. family’s view of their part of the ability in the family	E.g. family’s view of the family’s ability as a whole
Collect data from two (or more) persons and make separate analyses for the two dyad members (single-informant perspective)	E.g. patient’s and family member’s view of his/her ability in the family	E.g. patient’s and family member’s view of two family members’ ability in the family	E.g. patient’s and family member’s view of the family’s ability as a whole

To yield reliable and valid measures and be able to make accurate decisions about FSOC, there is a need for well-designed scales with satisfactory measurement properties. This study explores the measurement properties of the 12-item FSOC-S12 according to the Rasch model, from the dyadic- and single-informant perspectives.

## Methods

### Study design

This psychometric study used data from a previous research project regarding families’ life situations when living with cancer [7, 26]. This study included only complete dyads, with one patient and one family member. The Regional Ethical Review Boards in Linköping, Sweden, approved the study (No. 2014/70-31).

### Participants

Participants for the overall research project were recruited from two palliative centres and two oncology clinics in the south of Sweden, between May 2015 and October 2016. The care units were selected through convenience sampling in one large city, a mid-sized city, and two small towns. Patients recruited consecutively were Swedish-speaking, older than 18 years with a diagnosis of cancer in the palliative stage, and each patient invited one family member to participate. Family members were defined as individuals to whom the patient felt linked to via a sense of belonging and engagement in their lives (e.g., spouse, sibling, children or friend) [27]. In total, 179 patients and 165 family members were recruited and took part in the research project [7, 26]. For this study, only complete dyads including one patient and one family member from the same family were selected (*n* = 151).

### Procedure and data collection

Nurses at the palliative centres and oncology clinics distributed oral and written information regarding the study, and each patient and family member were asked to complete a study questionnaire. The questionnaire could be completed by paper-and-pencil or online. Paper based questionnaires were returned in a pre-paid envelope to the research group. The study questionnaire included demographic characteristics and the FSOC-S12 [15]. The FSOC-S12 is constructed as a unidimensional scale, including 12 items representing all three core components of SOC (i.e., comprehensibility, manageability, meaningfulness). An example of an FSOC-S12 item is: ‘To what extent does it seem to you that your family rules are clear to you?’. Responses were collected using a seven-point numerical rating scale [1–7] with item-specific anchor descriptors. The summed total score has a possible range between 12 and 84; higher scores imply a higher level of FSOC (15). In the Swedish version all items are scored in the same direction [26].

### Data analysis

Participants’ demographic characteristics and study variables were presented using descriptive statistics.

To assess the measurement properties of FSOC-S12 from a dyadic- and single-informant perspective, data were analysed according to the polytomous (partial credit) Rasch model [28] using Winsteps® 4.3.1 [29]. Details of the analyses are presented in Table 2 [30–34].

**Table 2:** Summary of measurement properties of the FSOC-S12, its interpretation from the initial analyses

Measurement property		Interpretation and criteria		FSOC-S12: DIP Initial analyses	FSOC-S12: DIP Analyses after collapsed thresholds	FSOC-S12: SIP Initial analyses	FSOC-S12: SIP Analyses after collapsed thresholds
Sample to item targeting	The relative distributions of item-threshold:person measures.	Mean person location close to zero [29]. Range of person locations Range of item-threshold locations		1.15 logits (SD 1.02) -0.76-5.98 -0.50-0.79	1.13 logits (SD 1.13) -1.81-6.02 -0.95-1.16	1.51 logits (SD 1.30) -0.85-5.89 -0.55-0.93	1.34 logits (SD 1.45) -2.52-5.83 -0.98-1.28
Response category function	Ratings should be consistent with the estimate of the underlying construct	Monotonically increasing thresholds [31].		4 of 24 had monotonically increasing thresholds	All had monotonically increasing thresholds	1 of 12 had monotonically increasing thresholds	All had monotonically increasing thresholds
Item fit to the model	How observed data accord with the Rasch model.	MNSQ is recommended to be within 0.5-1.5 and ZSTD is recommended not to exceed ±2SD [32].	MNSQ OUTFIT	23 of 24 in recommended range	23 of 24 in recommended range	All in recommended range	All in recommended range
			ZSTD OUTFIT	21 of 24 inside recommended range	22 of 24 inside recommended range	9 of 12 inside recommended range	11 of 12 inside recommended range
			MNSQ INFIT	All in recommended range	All in recommended range	All in recommended range	All in recommended range
			ZSTD INFIT	22 of 24 inside recommended range	23 of 24 inside recommended range	11 of 12 inside recommended range	11 of 12 inside recommended range
Unidimensionality	Whether items appear to represent one common variable	Eigenvalue of unexplained variance in the 1^st^ contrast of a PCA of fit residuals should not be greater than 2 [33].	Eigenvalue	3.44	3.60	1.71	1.75
		Disattenuated Pearson correlations of the person measures close to 1	Disattenuated Pearson correlation	0.61	0.62	1.00	1.00
Local independence (LD)	There should be no dependence between two items, i.e., item responses should be independent of each other	Relative correlations of residuals should not exceed the relative Q3* cut off [45]: - 0.24 for dyadic-informant perspective 0.12 for single-informant perspective		LD for 9 of 276 correlations	LD for 9 of 276 correlations	LD for 3 of 66 correlations	LD for 4 of 66 correlations
Differential item function (DIF)	DIF occurs when items behave different for differently sub-groups, in this paper, between patients and their family members from the single-informant perspective	There should be no statistical differences between item estimates, p-value should not be significant be below 0.05	DIF probability	N/A	N/A	Item 4: 0.01 Item 12: 0.01	Item 4: 0.02 Item 6: 0.03 Item 8: 0.03 Item 12: 0.01
		DIF ≥ 0.64 logits = moderate to large; DIF ≥ 0.43 = slight to moderate.	DIF size	N/A	N/A	Item 4: 0.36 Item 12: 0.32	Item 4: 0.42 Item 6: 0.33 Item 8: 0.51 Item 12: 0.42
Reliability	The proportion of variance that is true variance	0 implies all error and 1 implies no error	Person reliability	0.87	0.89	0.82	0.84
			Item reliability	0.95	0.97	0.98	0.99

^*^Response options 1 and 2 as well as options 3 and 4 were collapsed into two categories for all items, except for item 1 where response options 1, 2, 3 and 4 were collapsed into one category and for item 8 where response options 1, 2, 3 and 4 as well as response options 5 and 6 were collapsed into two categories.

*DIP* Dyadic-informant perspective, *SIP* Single informant perspective, *DIF* Differential item functioning, *INFIT* Inlier-pattern sensitive statistics, *LD* Local dependency, *MNSQ* Mean square fit statistics, *OUTFIT* Outlier-pattern sensitive statistics, *PCA* Principal component analysis, *ZSTD* z-standardized fit statistics

The Rasch model makes two main assertions: (i) the lower the location of an item, the more likely it will be affirmed, and (ii) the higher the location of the attribute an agent has, the more likely they will affirm an item. In turn, the Rasch model enables separate measures of the agent attribute (here the family’s ability to feel FSOC) and the item attribute (here the FSOC task difficulty) on a conjoint interval scale corresponding to the measurement continuum of the FSOC.

**Fig. 1:**
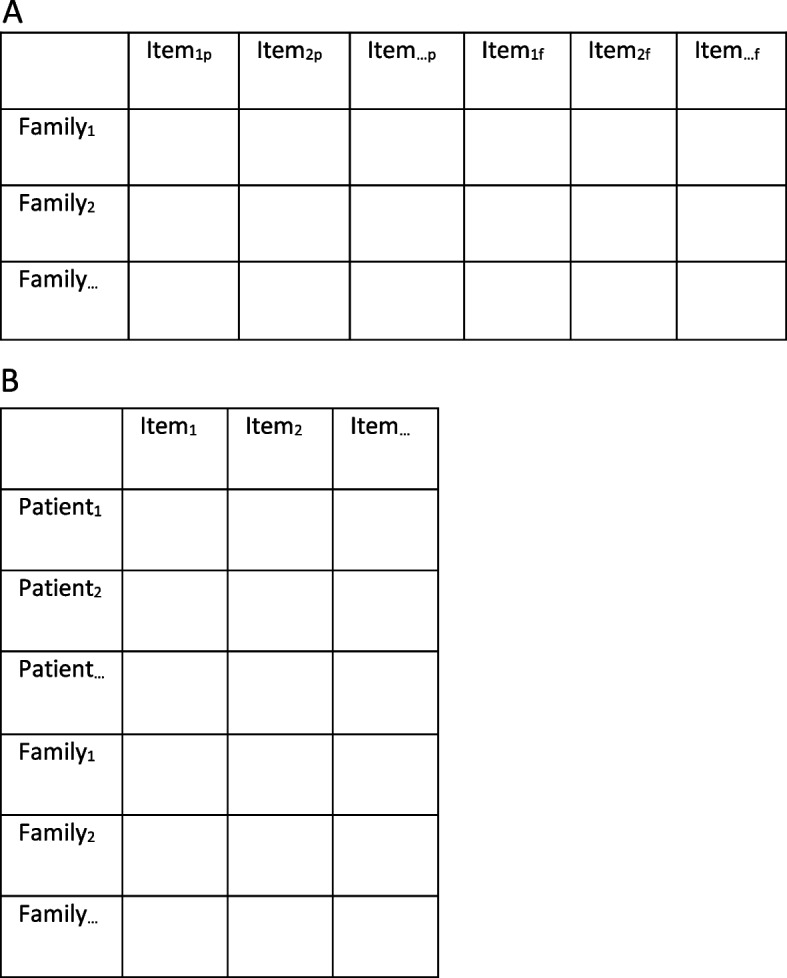
Conceptual visualization for (**A**) racked data (dyadic-informant perspective), and (**B**) stacked data, (single informant perspective)

Data were racked and stacked to assess the two different perspectives for this study, i.e. a dyadic- and single-informant perspective [35], as illustrated in Figure 1. Racking (Fig. 1a) refers to placing items for the patient and the family member together horizontally, thus providing a dyadic-informant perspective on the FSOC-S12 where each item is treated as two respondent-role specific items. This allows for separate estimates of FSOC task (item) difficulties for patients and family members, respectively. However, the Rasch model assumes that items are locally independent, but when the ‘same’ item is used twice, a potential risk of local dependence is apparent (similar to discussions by Andrich & Krainer [36], Andrich et al [37], Olsbjerg & Christensen [38]. Thus, local dependence needs to be examined. Stacking (Fig. 1b) refers to vertically placing items from patients and family members together, thus representing the single-informant perspective. When stacking data, item difficulties are assumed to be equal across respondent groups (which is formally tested by differential item function, DIF), but differences in dyad-member ability to feel FSOC are allowed. Stacking data also allows differences in perceived FSOC within the dyad (i.e., between patients and family members) to be assessed.

To further assess relations, similarities, and differences in FSOC-S12 between a dyadic- and a single-informant perspective, we:

I. Compared the hierarchical ordering of items and correlated (Pearson correlation) item measures from the different perspectives as well as plotted agreements (Bland–Altman plot).
II. Correlated (Pearson correlation) person measures from the patient and family members, plotted agreements (Bland–Altman plot), and compared (t-tests) individual person measures from patient and family members based on the single-informant perspective.

## Results

### Sample characteristics

In total, 151 dyads with one patient and one family member were included. The number of women and men was equally distributed among persons with cancer (51% vs. 49%), while there were more women than men among family members (64% vs. 36%). The mean age was 68.4 years for patients and 62.5 years for family members. The majority (69.5 %) of family members had a partner relationship to the patient (Table 3).

**Table 3:** Characteristics of the dyads (*n* = 151)

Variables	Patients with cancer, *n* = 151	Family members, *n* = 151
Sex, n (%)		
Male	77 (51.0)	54 (35.8)
Female	74 (49.0)	97 (64.2)
Age, mean (SD; min-max)	68.4 (10.0; 39-86)	62.5 (13.4; 15-91)
Education, n (%)		
Below primary school	3 (2.0)	1 (0.7)
Primary school	28 (18.5)	25 (16.6)
High school	65 (43.0)	59 (39.1)
University	55 (36.4)	66 (43.7)
Occupation, n (%)		
Employed	18 (11.9)	61 (40.4)
Student	0	1 (0.7)
Retired	106 (70.2)	82 (54.3)
Sick leave	22 (14.6)	4 (2.6)
Other	5 (3.3)	3 (2.0)
Monthly household income (euros), n (%)		
0-1,499	9 (6.0)	4 (2.6)
1,500-2,999	51 (33.8)	42 (27.8)
3,000-4,499	42 (27.8)	46 (30.5)
> 4,500	48 (31.8)	58 (38.4)
Missing	1 (0.7)	1 (0.7)
Diagnosis, n (%)		
Breast cancer	26 (17.2)	N/A
Colon cancer	23 (15.2)	N/A
Prostate cancer	18 (11.9)	N/A
Kidney cancer	21 (13.9)	N/A
Other cancers	63 (41.7)	N/A
Relation to the patient, n (%)		
Partner relationship	N/A	105 (69.5)
Children	N/A	33 (21.9)
Sibling	N/A	5 (3.3)
Friend	N/A	4 (2.6)
Parent	N/A	3 (2.0)
Niece	N/A	1 (0.7)
FSOC-S12, Md (q1–q3; min-max)	72 (65–77; 38–84)	70 (64–76; 48–84)

FSOC-S12 = Family Sense of Coherence Scale, 12-item short version

### Measurement properties of the FSOC-S12: A dyadic-informant perspective

Table 2 provides a summary of the analyses of both disordered and collapsed threshold of FSOC-S12 with a dyadic-informant perspective. The initial analyses revealed problems with reversed thresholds for all except four items. Ordered thresholds were obtained by collapsing response categories 1 and 2 and categories 3 and 4 into two categories for most items (items 2-7 and 9-12). Items 1 and 8 required further collapses; response categories 1, 2, 3 and 4 into one category for item 1 and 1, 2, 3 and 4 as well as response categories 5 and 6 for item 8 into two categories.

Table 4 gives the item measures and fit statistics from the revised analysis with resolved threshold ordering. The items are ordered from lower to higher measures, thus representing a hierarchy from the lowest to the highest item locations. It is clear that the ‘same’ items responded to by either the patient or the family member were located close to each other. For example, item 10 has the lowest location for both patients (-0.75; 2SE, 0.22) and family members (-0.95; 2SE, 0.24) and Item 1 has highest location for both patients (1.01; 2SE, 0.8) and family members (1.11; 2SE, 0.18)*.*

**Table 4:** FSOC-S12 item statistics with a dyadic-informant perspective following collapsing of thresholds

Item	Respondent	Item content (abridged)	Measure	2SE	MNSQ OUTFIT	ZSTD OUTFIT	MNSQ INFIT	ZSTD INFIT	Cluster*	Loading**
Item 10	Family member	Your role in the family is satisfactory	-0.95	0.24	0.86	-0.66	0.93	-0.46	1	0.45
Item 8	Patient	Feeling that you are being treated unfairly	-0.79	0.32	0.80	-1.01	0.94	-0.42	3	-0.30
Item 10	Patient	Your role in the family is satisfactory	-0.75	0.22	0.79	-0.83	0.93	-0.40	3	-0.28
Item 9	Family member	When you think about your family, you think how great it is to be alive	-0.75	0.2	0.96	-0.14	0.85	-1.21	1	0.4
Item 3	Family member	Your family life has had very clear goals	-0.62	0.22	0.94	-0.21	1.10	0.66	1	0.45
Item 3	Patient	Your family life has had very clear goals	-0.57	0.22	1.58	**2.14**	0.94	-0.33	3	-0.35
Item 8	Family member	Feeling that you are being treated unfairly	-0.56	0.32	1.03	0.25	1.00	-0.01	2	0.24
Item 4	Patient	Your family rules are clear to you	-0.51	0.2	0.82	-1.10	0.91	-0.60	3	-0.47
Item 9	Patient	When you think about your family, you think how great it is to be alive	-0.43	0.2	1.22	1.03	0.96	-0.23	2	-0.08
Item 2	Family member	The family’s ability to cooperate	-0.39	0.2	0.92	-0.50	0.86	-1.18	1	0.44
Item 2	Patient	The family’s ability to cooperate	-0.38	0.2	0.92	-0.48	0.94	-0.46	3	-0.40
Item 12	Patient	Have your family ever disappointed you	-0.26	0.2	1.00	0.07	1.08	0.54	3	-0.36
Item 4	Family member	Your family rules are clear to you	-0.22	0.22	0.88	-0.67	0.93	-0.53	2	0.25
Item 12	Family member	Have your family ever disappointed you	-0.03	0.2	1.18	1.43	1.19	1.63	2	0.23
Item 5	Patient	When your family faces a difficult situation	0.25	0.18	0.86	-1.26	0.88	-1.16	3	-0.47
Item 7	Patient	To what extent is the future of your family clear	0.48	0.16	0.94	-0.41	0.99	-0.03	3	-0.47
Item 6	Patient	Your family life seems to you: full of interest	0.54	0.2	1.03	0.30	1.06	0.63	3	-0.32
Item 6	Family member	Your family life seems to you: full of interest	0.56	0.18	1.03	0.36	1.06	0.62	1	0.36
Item 5	Family member	When your family faces a difficult situation	0.59	0.2	0.98	-0.15	1.00	-0.02	1	0.47
Item 7	Family member	To what extent is the future of your family clear	0.62	0.18	0.95	-0.40	1.00	0.01	1	0.51
Item 11	Patient	You have the feeling that you don’t know exactly what will happen in your family	0.92	0.18	1.34	**2.88**	1.28	**2.51**	3	-0.41
Item 1	Patient	Can you influence what happens in your family	0.99	0.18	1.26	1.90	1.16	1.56	3	-0.43
Item 1	Family member	Can you influence what happens in your family	1.11	0.18	1.14	1.04	1.01	0.16	1	0.36
Item 11	Family member	You have the feeling that you don’t know exactly what will happen in your family	1.16	0.2	1.01	0.16	1.01	0.17	1	0.48

^*^Clusters and ** loadings are derived from principal component analysis (PCA) of the fit residuals used to assess unidimensionality. Items are ordered from easiest at the top to the most challenging at the bottom (i.e., according to the measure column).

2SE = 2 standard error, corresponding ~95% CI INFIT = Inlier-pattern sensitive statistics; MNSQ = Mean square fit statistics; OUTFIT = Outlier-pattern sensitive statistics; ZSTD = Z-standardized fit statistics. Bolded numbers indicate misfit, i.e., ±2SD.

Two items (items 3 and 11) demonstrated misfit among the patients; both showed OUTFIT ZSTD values outside the expected range, and item 11 also exhibited a larger INFIT ZSTD than expected (Table 4). No DIF by role was detected in the dyad, i.e., patient vs. family member (Table 4).

The eigenvalue of unexplained variance in the first contrast was 3.60 and, by examining the three item clusters derived from the loadings in the principal component analysis (PCA) of the fit residuals, it was evident that items were mainly grouped according to the two respondent groups, i.e., patients in cluster 3 and family members in cluster 1 and 2 (Table 4).

Problems with local dependency were identified when residual correlations were above the relative Q3* cut-off (0.24) as found within the dyads (i.e., residual correlations between patients and family members) for item 8 (0.33) and item 9 (0.41). Furthermore, local dependencies were identified for patients between items 7 and 11 (0.26), and for family members between items 2 and 3 (0.29), 3 and 4 (0.26), 5 and 6 (0.25), 3 and 10 (0.27), 9 and 10 (0.37), and 7 and 11 (0.42).

### Measurement properties of the FSOC-S12: A single-informant perspective

Table 2 summarises the analyses of both disordered and collapsed threshold of FSOC-S12 from a single-informant perspective. Regarding the analysis from a single-informant perspective, disordered thresholds were present for all items except item 7. Thus, response categories were collapsed using the same strategy as that for the dyadic-informant perspective.

Table 5 provides the item measures and fit statistics from the revised analysis with resolved threshold ordering. As in Table 4, items in Table 5 are ordered from lower to higher measures and the item hierarchy is similar for the analyses of a single-informant perspective compared with a dyadic-informant perspective. This is further elaborated below.

**Table 5:** Item fit statistics for FSOC-S12 analysis of a single-informant perspective when disordered thresholds are resolved

Item	Item content (abridged)	Measure	2SE	MNSQ OUTFIT	ZSTD OUTFIT	MNSQ INFIT	ZSTD INFIT	Cluster *	Loading **	Concepts ***
Item 10	Your role in the family is satisfactory	-0.98	0.18	0.88	-1.12	0.85	-0.86	3	-0.60	Meaningfull
Item 4	Your family rules are clear to you	-0.88	0.16	0.95	-0.52	0.91	-0.74	3	-0.42	Comprehensible
Item 3	Your family life has had very clear goals	-0.74	0.18	0.91	-0.75	1.03	0.23	3	-0.47	Meaningfull
Item 8	Feeling that you are being treated unfairly	-0.69	0.24	0.91	-1.12	0.87	-1.07	2	-0.05	Manageable
Item 9	When you think about your family, you think how great it is to be alive	-0.63	0.16	0.95	-0.43	1.28	1.74	3	-0.32	Meaningfull
Item 2	The family’s ability to cooperate	-0.43	0.16	0.91	-1.06	0.87	-1.16	2	-0.02	Manageable
Item 12	Have your family ever disappointed you	-0.16	0.14	1.18	**2.05**	1.21	**2.08**	2	-0.19	Manageable
Item 5	When your family faces a difficult situation	0.55	0.14	0.88	-1.58	0.87	-1.71	2	0.20	Manageable
Item 7	To what extent is the future of your family clear	0.69	0.14	0.89	-1.35	0.86	-1.55	1	0.63	Meaningfull
Item 6	Your family life seems to you: full of interest	0.71	0.14	1.16	1.92	1.13	1.63	1	0.40	Comprehensible
Item 1	Can you influence what happens in your family	1.27	0.14	1.10	1.23	1.20	1.83	2	-0.15	Comprehensible
Item 11	You have the feeling that you don’t know exactly what will happen in your family	1.28	0.14	1.09	1.14	1.13	1.64	1	0.48	Comprehensible

^*^Clusters and ** loadings are derived from the principal component analysis (PCA) of the fit residuals used to assess unidimensionality. *** Concepts refer to the original proposal of the three components (i.e., meaningful, manageable, and comprehensible) that constitutes SOC from Antonowsky (1987). Items are ordered from easiest at the top to the most challenging at the bottom (i.e., according to the measure column)s

*2SE* 2 standard error, corresponding ~95%,CI, INFIT = Inlier-pattern sensitive statistics, *LD* Local dependency, *MNSQ* Mean square fit statistics, *OUTFIT* Outlier-pattern sensitive statistics, *ZSTD* Z-standardized fit statistics. Bolded numbers indicate misfit, i.e., ±2SD

Only item 12 demonstrated misfit (OUTFIT ZSTD value 2.20). The eigenvalue of the unexplained variance in the first contrast was 1.75, and the correlation between clusters 1 and 3 was 1.00. As shown in Table 5, the clusters did not fully correspond to the three concepts proposed by Antonowsky [6], and the three concepts were not strictly hierarchically ordered, although, items stemming from meaningfulness tended to be easier, followed by more challenging items from manageability and the most challenging items from comprehensibility (Table 5).

Four items showed significant DIF by role of the informant. As shown in Table 2, item 8 showed a slight to moderate DIF by role with a slightly higher estimate of task difficulty from family members than from patients, while the DIF size was smaller for items 4, 6 and 12. For item 12 the family members had the highest task location, while for items 4 and 6 patients had the highest task location.

### Comparisons between the dyadic- and single-informant perspectives

**Fig. 2:**
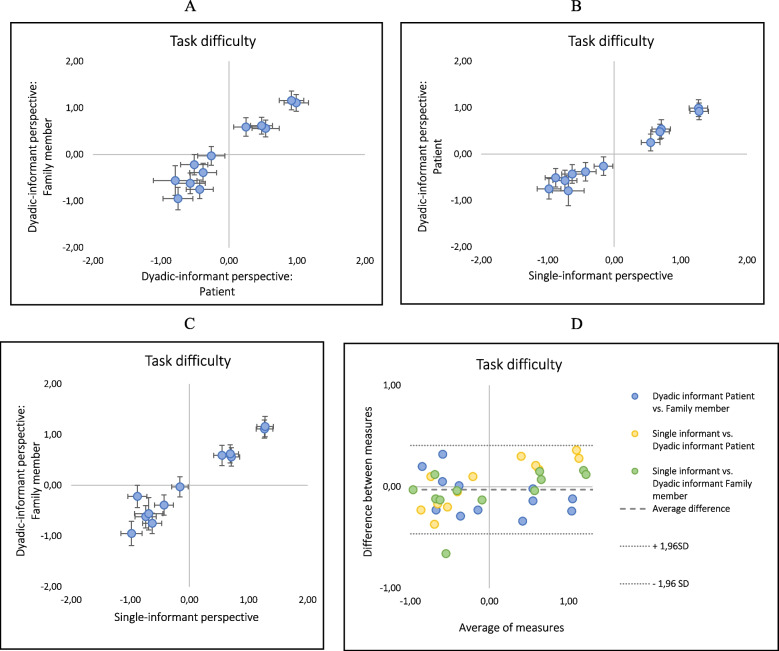
Pearson correlation plots for task difficulty estimates based on (**A**) family members vs. patients in analyses with a dyadic-informant perspective, (**B**) patients from a dyadic-informant perspective vs. a single-informant perspective, and (**C**) family members from a dyadic-informant perspective vs. a single-informant perspective. Error bars indicate 2SE. Plotted agreements (**D**) Dyadic informant patient vs family member, single informant vs dyadic informant patient and single informant vs dyadic family member

As described above and presented in Tables 4 and 5, the item hierarchy is similar when comparing analyses based on single- and dyadic-informant perspectives. This is further illustrated in Figures 2A-C, where measures of task location based on the different perspectives and respondents are plotted. The Pearson correlation coefficients ranged from 0.96 to 0.97. Item 4 deviated from the hierarchical structures when comparing the item measure from the family members from the dyadic-informant perspective with the item measure from single-informant perspective, which is shown as an outlying dot in Figure 2C. Thus, estimation of task location for item 4 based on family members from the dyadic-informant perspective set up (y-axis) was higher compared with when estimated from the single-informant perspective (x-axis).This result is also corroborated in the Bland-Altman plot (Figure 2D), showing all items within +/-1.96SD except item 4 when comparing family members from the dyadic-informant perspective with the single-informant perspective.

**Fig. 3:**
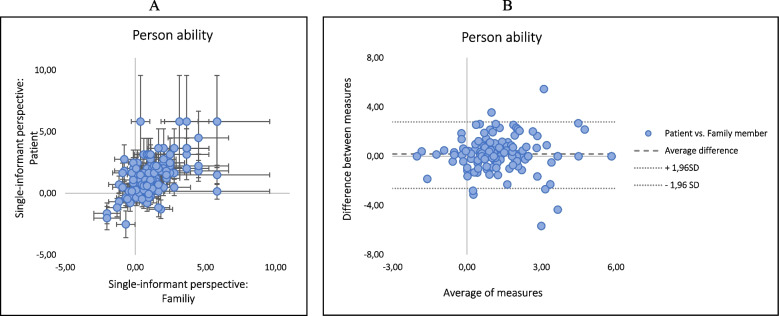
Correlations of person ability estimates within the dyads from a single informant perspective (**A**) and the comparisons in a Bland-Altman plot (**B**)

Person locations correlated moderately (0.58) between patients’ and family members’ ratings from a single-informant perspective (Figure 3A) and almost all comparisons where within +/-1.96SD in the Bland-Altman plot (Figure 3B). In seven cases (4%), the patient showed a significantly lower measure than the family members and part of this was reflected in person fit statistics (e.g., three dyads with INFIT ZSTD > 2SD). In contrast, 11 (7%) family members showed a significantly lower measure than the corresponding patients and five of those dyads had INFIT ZSTD > 2SD.

## Discussion

To the best of our knowledge, this is the first evaluation of the FSOC-S12 using the Rasch model and from a dyadic analytical approach. The choice of the Rasch model in favour to any other model, more commonly used in studies with dyadic data [25], in line with a measurement science perspective, rather than data modelling [19, 39]. We have thus advanced previous work, on the FSOC-S12 by applying the Rasch model, which enable linear measurement based on ordinal observations, separation of person and item attributes, and detailed insights on the measurement properties. Overall, the FSOC-S12 demonstrated minor deviations from the Rasch model among dyads of patients and family members in palliative cancer care. Furthermore, our findings provide novel insights into the FSOC construct and how to measure it from different perspectives using the FSOC-S12. Both data set-ups are useful, and the choice depends on the clinical or research question, the FSOC-S12 can be used to measure the family’s overall perception of FSOC (dyadic-informant perspective) or to measure the patient’s and family member’s individual perceptions of FSOC (single-informant perspective).

Despite the conceptual difference between the dyadic- and single-informant perspectives, the item hierarchy ordering appears to be similar. This provides further support for the construct validity of the FSOC-S12 and could be the start of a coherent construct theory of what less to more sense of coherence in the family means. Practically, this implies that the item hierarchy in FSOC-S12 can inform where a family is located on the continuum and provides clinicians and families with a ‘compass’, pointing the way forward for actions needed [40].

Additional key questions are whether a single-informant perspective is enough to measure FSOC, and what is measured when only one party gives his or her voice about the family as a whole. A single-informant perspective does not consider that the whole family’s experiences are more than the sum of each family member’s experiences [27, 41] and that the family´s reality can be understood as multidimensional, which means that family members’ different descriptions of the same situation may be equally valid [42]. However, the dyadic informant perspective indicates some dimensionality issues with the FSOC-S12. Multidimensionality always exists to some extent [43, 44], although the critical question is whether it is significant enough [45]. At this stage we can only speculate about this; the dimensionality issues may be a consequence of a lack of agreement within families and could be explained in terms of the FSOC referring to different constructs for patients and family members. Furthermore, in the present study, we only included two informants in the family dyads, and one may ask if more informants are needed to provide a reliable measure for the family as a whole [27, 41]. With only two informants in some families, all members have not had the chance to raise their voice.

From a dyadic-informant perspective, the same statement is repeated twice: once when the patient responds and once when the family member responds. It is, therefore, likely that patients’ and family members’ responses are dependent upon each other, thus causing local dependency within the item-pair [36–38]. In this study, local dependency was, however, only apparent for two items within the dyads: item 8 [Do you have the feeling that you are being treated unfairly by your family?] and item 9 [When you think about your family, you very often feel how great it is to be alive]. While there are more sophisticated methods for investigating and accommodate local dependencies [36–38, 46, 47] , this suggest further qualitative investigations on why local dependency only was found for items 8 and 9 within the dyads and not for the other items. It should also be noted that local dependency is based on fit residuals [48, 49], which is affected by the sample size [50, 51]. Thus, at the present stage with a somewhat small sample size, we recommend a mixed-method approach combining statistical results from a larger sample size with the qualitatively meaning of the items from patients and family members, respectively, to better understand local dependency issues when applying a dyadic-informant perspective on FSOC.

Given the different roles of patients and family members, items may be interpreted differently between these groups in the same way as it might differ between groups of different sex and age and potential DIF may suggest a lack of measurement invariance. In this study, DIF was found present in four items when comparing patients and family members. At the same time, the consequences on person measures were almost negligible. This warrants further exploration and at present we can only speculate about why DIF was only present in some items. DIF may be due to informal roles in the dyads [3, 52], e.g., if one partner has the power to stipulate rules in the family and therefore scores higher on item 4 [Your family rules are clear to you] and on item 8 [Feeling that you are being treated unfairly]. The DIF may also be due to if one of the dyads had a good day and the other one had a bad day, e.g., if one partner has a bad day, therefore, they score lower on item 6 [Your family life seems to you: full of interest]. In further studies, we encourage deeper investigations on the relation between the patient and family member and disease severity, which also warrants larger samples, to get a clearer picture of DIF.

## Methodological considerations

There are some methodological consideration with the present study to bear in mind. First, 151 dyads can be considered to be a small sample [53]. One consequence is that it may have affected threshold ordering negatively due to the few respondents using each response category [54]. Disordered thresholds may have other explanations, for example that only the extreme response categories are labelled [55] and difficulties differentiating between seven levels [56]. Therefore, further studies with larger samples are needed to better understand potential problems with model fit, DIF and local dependency [50, 51] and before any firm conclusions can be drawn. The small sample is a consequence of the fact that the present study is based on data from a previous study that was not designed to address dyadic data analyses. Another limitation is that the type of relationship between the patient and family member has not been considered in the present study. The reason was that a vast majority were partners. The design of the present study did not allow any drop out analysis. Therefore, we cannot exclude any type of attrition bias. There is signs of attrition bias due to socioeconomic status since a large share of the participants had a university degree and a high income level. Even if this is a threat for the external validity, it is of minor importance for the psychometric properties. This is particularly true for the Rasch model which is sample independent in contrast to models under classical test theory. Therefore, the result should be carefully generalised. However, the insights from this study can be of value to better understand and improve the FSOC-S12 as well as for designing future studies using dyadic data.

There is a risk that items are regarded as misfitting due to too large sample sizes (type I error), and conversely, there is a risk of not identifying misfitting items correctly with too small sample sizes (type II errors). Those risks in relation to sample size are also affected by weather conditional or unconditional infit or outfit statistics are used [57]. Winsteps, which was used in the present study, provides unconditional models which may be associated with inflated type I error rates at sample sizes of 250-500 or more [57]. While our sample is not associated with any obvious risks unreliable fit statistics, those results should be interpreted with some caution.

## Conclusion

Our approach to handling dyadic data has shown both strengths and limitations in the evaluation of FSOC-S12. This study provides important insights into the dyadic- and single-informant perspectives when using the FSOC-S12 in family research. Notably, both perspectives showed minor deviations from the Rasch model. Depending on the clinical or research question to respond, at present, the FSOC-S12 may be used to provide meaningful measures of family’s view of the family ability as a whole (dyadic-informant perspective) or measures of patient’s and family member’s own view of the family ability as a whole (single-informant perspective). However, we encourage further studies to consider three closely related conceptual and methodological aspects, (i) conceptual differences between the dyadic- and single-informant perspectives, (ii) if a single-informant perspective is enough to measure family as a whole, and (iii) if more than two informants are needed to measure the family as a whole.

## Data Availability

The datasets generated and analysed during the current study are not available for public use, due to confidentiality, but are available from the corresponding author on reasonable request.

## Abbreviations

FSOC: family sense of coherence
FSOC-S12: Family sense of coherence scale 12 items
FSOC-S26: Family sense of coherence scale 26 items
DIF: differential item functioning
INFIT: Inlier-pattern sensitive statistics
LD: Local dependency
MNSQ: Mean square fit statistics
OUTFIT: Outlier-pattern sensitive statistics
PCA: principal component analysis
SE: Standard error
SOC: Sense of coherence
SOC-S: Sense of Coherence Scale
ZSTD: z-standardized fit statistics
